# Continuous versus discrete quantity discrimination in dune snail (Mollusca: Gastropoda) seeking thermal refuges

**DOI:** 10.1038/s41598-021-82249-6

**Published:** 2021-02-12

**Authors:** Angelo Bisazza, Elia Gatto

**Affiliations:** 1 Department of General Psychology, University of Padova, Padua, Italy; 2 Padova Neuroscience Center, University of Padova, Padua, Italy

**Keywords:** Ecology, Zoology

## Abstract

The ability of invertebrates to discriminate quantities is poorly studied, and it is unknown whether other phyla possess the same richness and sophistication of quantification mechanisms observed in vertebrates. The dune snail, *Theba pisana*, occupies a harsh habitat characterised by sparse vegetation and diurnal soil temperatures well above the thermal tolerance of this species. To survive, a snail must locate and climb one of the rare tall herbs each dawn and spend the daytime hours in an elevated refuge position. Based on their ecology, we predicted that dune snails would prefer larger to smaller groups of refuges. We simulated shelter choice under controlled laboratory conditions. Snails’ acuity in discriminating quantity of shelters was comparable to that of mammals and birds, reaching the 4 versus 5 item discrimination, suggesting that natural selection could drive the evolution of advanced cognitive abilities even in small-brained animals if these functions have a high survival value. In a subsequent series of experiments, we investigated whether snails used numerical information or based their decisions upon continuous quantities, such as cumulative surface, density or convex hull, which co-varies with number. Though our results tend to underplay the role of these continuous cues, behavioural data alone are insufficient to determine if dune snails were using numerical information, leaving open the question of whether gastropod molluscans possess elementary abilities for numerical processing.

## Introduction

Continuous and discrete quantity information are important in guiding animal behaviour in virtually all aspects of life. The capacity to evaluate continuous (uncountable) quantities, such as length, area, weight, or duration, is widespread and can be found in organisms with relatively simple nervous systems, such as annelids, crustaceans, or gastropds^1–3^. This quantitative information takes part in decision-making processes in different contexts. For example, animals may gauge body sizes of rivals or prospective mates, assess distances from home, or estimate the extent of a food patch^4–6^.

Several vertebrates, from teleost fishes to primates, can also process discrete (countable) information. For example, many species are capable of accurately estimating the number of elements in a set and comparing the numerosity of different sets^7,8^. Studies conducted in nature or in the laboratory have shown that numerical abilities serve important adaptive functions. For example, in guppies, New Zealand robins, and macaques, quantity discrimination is used to select the patch containing the larger number of food items^9–11^. Conversely, some predators, namely lions and striped field mice, use this ability to select the smallest prey groups because they are more vulnerable to predation^12,13^. Various group-living mammals, including chimpanzees, lions, and hyenas, gauge the relative number of opponents before deciding whether to attack or withdraw^14–16^. Gregarious fish use the same ability to select the social group that provides the best protection from predators^17–19^. Some species, including eastern mosquitofish, brown-headed cowbirds, and American coots, use their quantitative abilities to increase reproductive success^20–22^.

Cognitive psychologists have shown that in these cases it is not necessary to assume the existence of a true numerical estimation system because an animal can use continuous cues, such as the amount of movement, the cumulative surface occupied by items, or the convex hull of the set as a proxy for number^23,24^. Inferring the existence of a numerical system requires a series of careful laboratory control experiments in which the animal is subjected to numerical tasks, while the access to non-numerical information is simultaneously prevented^8,25^. This process is not always straightforward and studies often fail to reach a firm conclusion even after numerous experiments are performed. In fact, convincing evidence of the presence of a numerical system exists only for a small fraction of the species investigated (e.g., guppy^26^, chicken^27^, and rhesus monkeys^10^).

It is not known whether numerical abilities have similar selective advantages in other phyla and whether numerical systems are widespread outside the vertebrate group. To date, this issue has been investigated only in a handful of species, and there is convincing evidence of a true numerical system for only one of them, the honeybee^28–30^. Honeybees, *Apis mellifera*, can be trained to discriminate different numbers of dots to obtain a food reward^31,32^. They are able to accomplish this task even when main continuous cues are controlled, thus it is suggested that they possess a numerical system analogous to that of vertebrates. Honeybees can also use ordinal information and learn the correct position in a sequence of artificial flowers when distance cues are made irrelevant^33^. Similar evidences have been recently provided for another social bee, the bumblebee, *Bombus terrestris*^34,35^. The function of cardinal and ordinal numerical abilities in social bees is unclear, but it has been suggested that they mainly serve to recognise flowers from the number of petals and to learn the location of food around their hives, respectively.

Circumstantial evidence suggests the ability to estimate the quantity of conspecifics in three other arthropod species. The juvenile spiders of *Portia africana* have been reported to take into account the number of competitors present when choosing between two patches of food^36^. Males of the coleopteran *Tenebrio molitor* are able to discriminate different numbers of females based on the odours they emit^37^. Ants (*Formica xerophila*) perceiving themselves as part of a large group are more aggressive towards another species than ants perceiving themselves as isolated individuals^38^. Controls are difficult to perform in these types of experiments, and it is unknown whether these three species are actually estimating the number of individuals or they are using other types of information as a proxy of number.

Recently, a mollusc, the cuttlefish, was observed to prefer the larger quantity of shrimps up to 4 versus 5 items^39^. Although authors manipulate some continuous cues (i.e., density and total activity of preys), it is unclear whether cuttlefish are really counting prey or are using other cues, such as the cumulative area occupied by shrimps or the convex hull of the groups.

**Figure 1:**
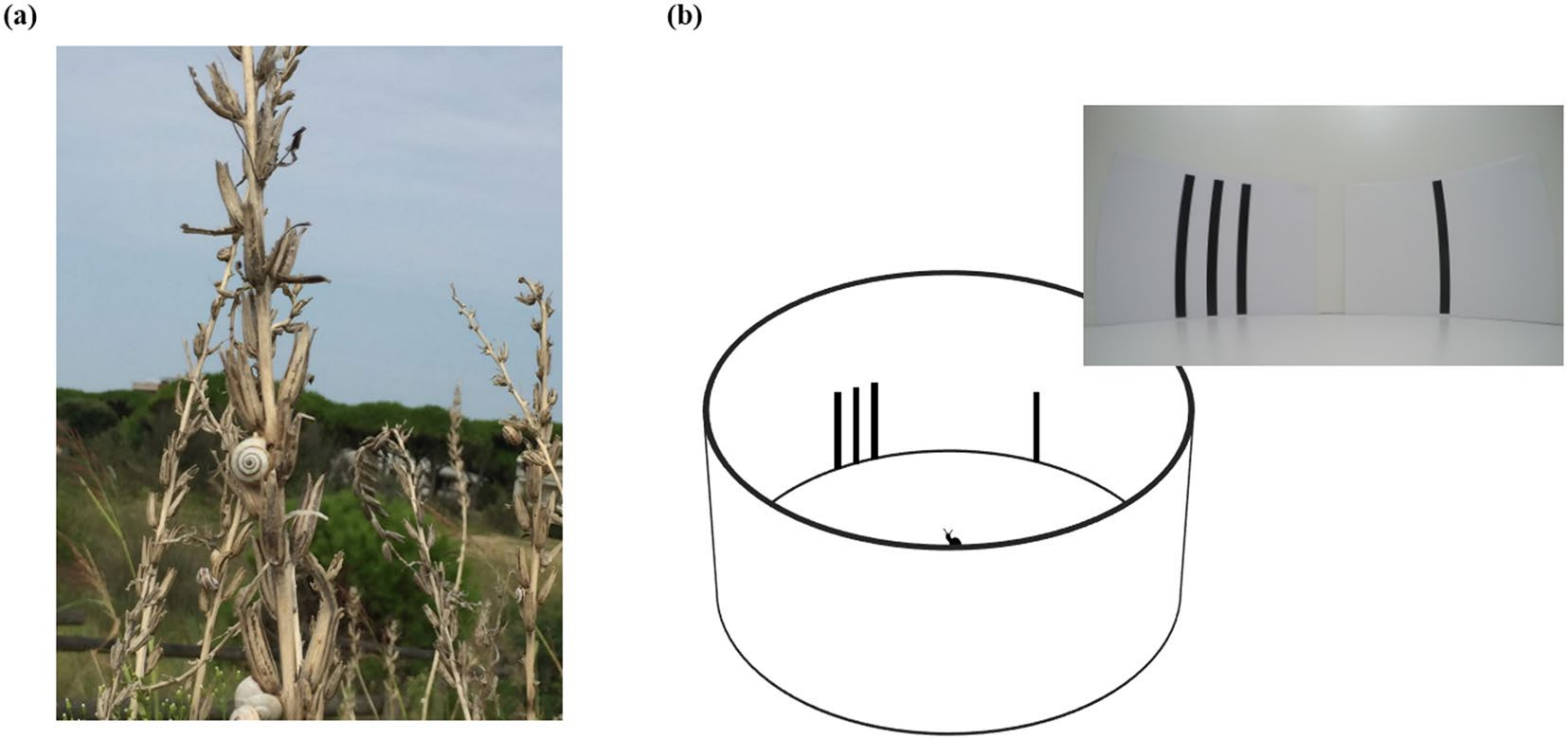
(**a**) Example of a dune snail *T. pisana* climbing on the stem of tall vegetation. (**b**) The circular arena used for investigating quantity discrimination ability in laboratory.

*Theba pisana* is a small terrestrial snail inhabiting the dunes of the Mediterranean coasts. Similar to most snails, it is active mainly at night. This species has a considerable thermal tolerance, with an upper lethal limit that lies, depending on exposure time, between 46 °C and 50 °C^40^. However, during sunny days, the sandy ground can reach temperatures that largely exceed this lethal limit (up to 75 °C). To avoid these adverse conditions at sunrise, dune snails climb the stem of tall vegetation, where the temperature rarely exceeds 30 °C, and remain inactive until night. If placed on the ground during the day, these snails rapidly regain an elevated position by orienting towards nearby stems and climbing on them (Fig. 1a; Supplementary Video S1). At our site of capture, snails were collected mainly from vertical, unbranched stems of live or dead inedible plants and herbs (e.g., *Puccinellia palustris*, *P. distans*, and *Juncus maritimus*).

Zanforlin showed that it is possible to simulate this behaviour in the laboratory^41^. After placing dune snails on a brightly lit arena, they rapidly orient towards a black cardboard shape on a white background and climb on it. With this setup, it was possible to study shape preference by placing two shapes at 60° angle from the centre and releasing the snail from the centre of the arena. He found that, confronted with similar geometric figures (e.g., two rectangles), snails oriented consistently towards the stimulus with the largest area. When area was kept constant, no particular preference for shape was observed, although there was a tendency to prefer the figure with a longer perimeter or with wider axes.

In all the experiments of the former study, snails were required to choose between two single shapes. In nature, however, stems are frequently arranged in clusters. All things being equal, there are several potential advantages in heading towards a large cluster of stems. In a cluster, there is greater probability of finding the stem with the most suitable features, such as a correct diameter or an optimal orientation to shelter from wind and sun^40^. In addition, not all the stems are accessible due to the presence of intricate or thorny vegetation at the base. Heading towards a group of stems increases the chances that at least one stem can be reached and climbed. Furthermore, most predators (mainly passerine birds, wall lizards, and rats) are small and catch only one or few preys at a time, and hence, sheltering in clusters could determine a dilution effect on predation risk^42,43^.

Based on the above considerations, we made the prediction that natural selection in *T. pisana* should favour the ability to discriminate between a single stem and a cluster and discriminate among clusters, based on the quantity of stems. The aim of the first experiment was to test this hypothesis. In the laboratory, we simulated stems used by dune snails as refuges by using black vertical bars on a white background (Fig. 1b; Supplementary Video S2). As we found that dune snails discriminate rather accurately between quantities of stems, in a series of subsequent experiments, we investigated the mechanism involved. Specifically, we tried to figure out if snails were using a true numerical system or if they used continuous quantitative information that co-varied with numerosity, such as the cumulative area occupied by items, their density or the convex hull they spanned.

### Experiment 1: discrimination of the quantity of refuges

**Figure 2:**
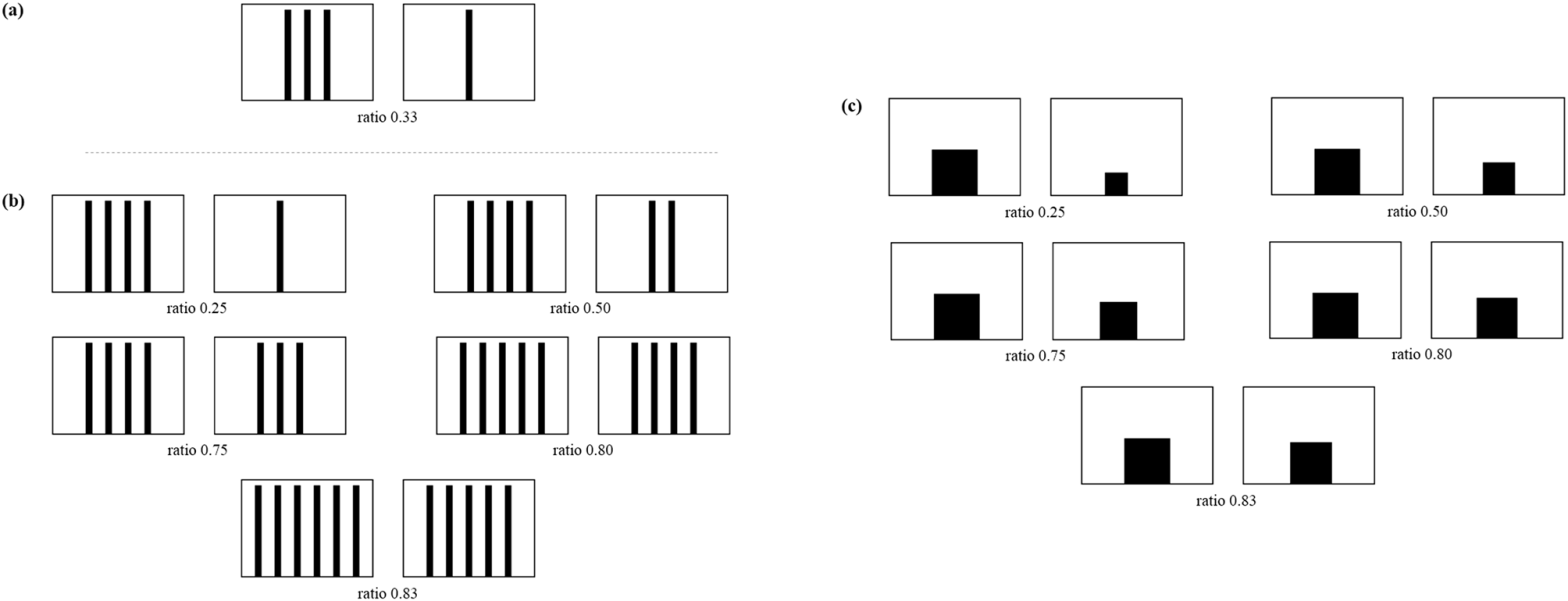
(**a**–**c**) Stimuli used in Experiment 1a, 1b and 1c, respectively.

**Figure 3:**
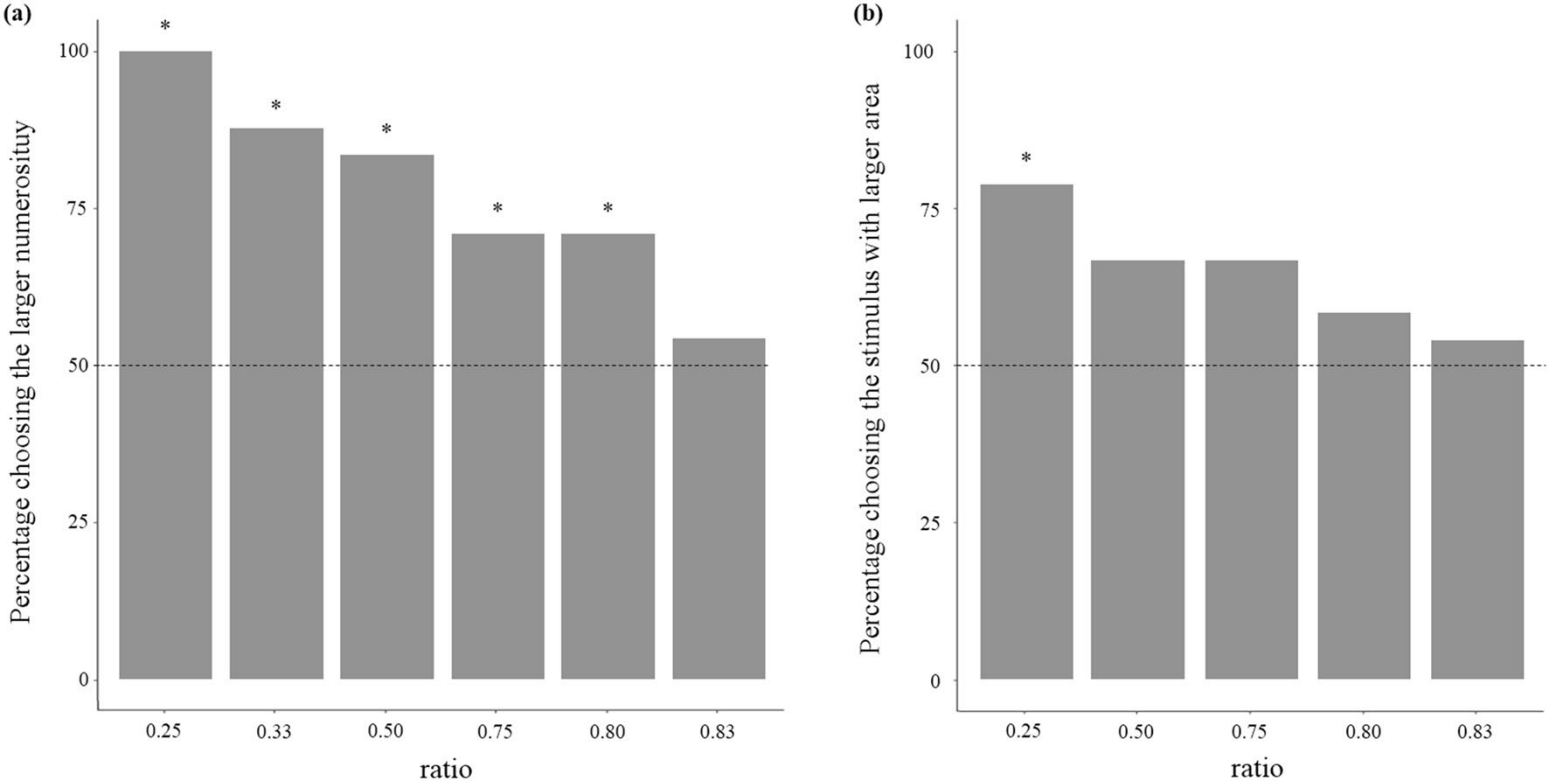
(**a**) Percentage of snails choosing the stimulus with larger quantity of bars in Experiment 1a and 1b. Snails showed a significant preference for larger quantity up to 4 versus 5 bars. There was a significant difference amongst the numerical ratios (*P* < 0.001). Dotted line represents the expected preference by chance, and asterisks indicate significant deviations from the chance level (*P* < 0.05). (**b**) Percentage of snails choosing the stimulus with larger area in Experiment 1c. Snails showed a significant preference for the larger stimulus only in the easiest discrimination (ratio 0.25; *P* = 0.004). Dotted line represents the expected preference by chance, and asterisks indicate significant deviations from the chance level (*P* < 0.05).

A previous study on dune snails investigated the choice between single objects that differed in shape and size^41^. However, based on their ecology, we predict that snails searching for protection from the heat also should focus on number and should move towards the largest available group of stems. In Experiment 1a, we studied whether dune snails prefer a group of refuges to a single one (Fig. 2a), and in Experiment 1b, we measured their accuracy to discriminate among groups of refuges differing in numerosity (Fig. 2b). To obtain reference data about snails’ general discriminatory abilities, in Experiment 1c, we measured the accuracy of dune snails to discriminate two equally shaped objects that differ in surface area (Fig. 3c).

#### Results and discussion

In Experiment 1a, 21 subjects of 24 chose the stimulus with three bars (*χ*^2^_1_ = 13.500, *P* < 0.001, effect size: *φ* = 0.530; Fig. 3a). In Experiment 1b, all 24 subjects chose the larger quantity in the 1 versus 4 discrimination (*χ*^2^_1_ = 24.000, *P* < 0.001, *φ* = 0.707), 20/24 subjects in 2 versus 4 (*χ*^2^_1_ = 7.538, *P* = 0.006, *φ* = 0.396), 17/24 in 3 versus 4 (*χ*^2^_1_ = 4.167, *P* = 0.041, *φ* = 0.295), and 17 /24 in 4 versus 5 (*χ*^2^_1_ = 4.167, *P* = 0.041, *φ* = 0.295); but 13 out of 24 subjects chose the larger quantity in 5 versus 6 (*χ*^2^_1_ = 0.167, *P* = 0.683, *φ* = 0.059). The general linear model (GLM) showed a significant difference among the numerical ratios (*χ*^2^_4_ = 21.790; *P* < 0.001, *R*^2^ = 0.215). We found a significant correlation between the numerical ratio and the degree of preference (Kendall non-parametric correlation on the six ratios of Experiments 1a and 1b: τ = 0.966; *P* = 0.007).

In Experiment 1c, snails significantly discriminated the stimulus with larger surface area in the easiest, 0.25-ratio area (19 out of 24 subjects; *χ*^2^_1_ = 8.167, *P* = 0.004, *φ* = 0.412; Fig. 3b). In the remaining four ratios, the number of subjects who chose the stimulus with larger surface area did not significantly differ from chance: 16/24 in the 0.50 ratio (*χ*^2^_1_ = 2.667, *P* = 0.103, *φ* = 0.236), 16/24 in the 0.75 ratio (*χ*^2^_1_ = 2.667, *P* = 0.103, *φ* = 0.236), 14/24 in the 0.80 ratio (*χ*^2^_1_ = 0.667, *P* = 0.414, *φ* = 0.118), and 13/24 in the 0.83 ratio (*χ*^2^_1_ = 0.167, *P* = 0.683, *φ* = 0.059). The general linear model (GLM) did not show a significant difference among the numerical ratios (*χ*^2^_3_ = 2.507; *P* = 0.474, *R*^2^ = 0.036). To contrast the accuracy in the numerical and the surface area discrimination, we performed an overall GLM analysis comparing the results of these two experiments for the ratios of 0.25–0.80. The 0.83 ratio was not included in the analysis since it was above the discrimination threshold in both experiments and hence non-informative. We found a significant effect of the ratio (*χ*^2^_4_ = 10.255, *P* = 0.017), a significant effect of the experiments (*χ*^2^_1_ = 4.908, *P* = 0.027), and no interaction (*χ*^2^_1_ = 6.601, *P* = 0.158, *R*^2^ = 0.148).

As predicted from their ecology, the large majority of dune snails preferred to approach a group of stems, instead of a single one. When we investigated their discrimination ability, we found snails to be surprisingly accurate in selecting the larger group. Preference is significant up to 4 versus 5 items, while preference drops to chance level in the comparison 5 versus 6. It is interesting to note that only primates and a few other vertebrates appear similar or more accurate in discriminating discrete quantities (chimpanzees^44^, rhesus monkeys^5^, and pigeons^45^), while many other species show much lower numerical acuity (e.g., red-backed salamander, 2 vs 3^46^; horses, 2 vs. 3^47^; and angelfish, 2 vs. 3^17^).

As for other cognitive skills, numerical abilities are commonly believed to correlate with the size and the degree of complexity of the nervous system^48–51^. However, one would expect natural selection to favour the evolution of specialised cognitive abilities, even in species with relatively simple brains, if these functions have high survival value^52,53^. Each day during summer, for a snail living along the coastal dunes, the probability of surviving until the next day crucially depends on its capacity to reach an adequate shelter. It is not surprising that this severe selective pressure could have promoted the evolution of elaborate mechanisms for shelter seeking, which include an extraordinary ability to estimate the quantity of available refuges in a cluster and make comparisons among clusters.

Various vertebrates and some invertebrates are able to perform tasks of this kind by using numerical information only. Yet, the result of our experiment does not necessarily imply that snails possess the capacity to process number. As we used the same type of bar for all stimuli, larger groups had a larger cumulative surface area and snail could have used this cue to orient toward the larger set. Among vertebrates the capacity of estimating areas, alone or in combination with other cues, can account for astonishing discrimination abilities. For example fish can discriminate the numerosity of two groups of conspecifics up to a 0.85 ratio relaying on cumulative area of the fish or on the amount of their movements; they do not use numerical information in this social context although they appear able to do so in other conditions^53^. An even simpler mechanism that snails could have used is scototaxis, i.e. the tendency to orient toward a dark part of the environment. Scototactic responses are widespread among invertebrates, and it was frequently observed that larger dark areas are preferred over small ones^54,55^.

This hypothesis seems to be supported by the results of Experiment 1c in which dune snails responded in a comparable way when tested in a discrimination of areas of two single shapes. It should be noticed that, contrarily to the expectancy of the cumulative area hypothesis, snails were significantly less accurate in discriminating two objects than two groups of objects. However, the difference in accuracy is small and might be explained by other factors, for example that snails were less motivated to choose the larger area due to the shape of the stimuli that differed substantially from that of a stem. In addition, subjects could have attended only to the height or to the width of stimuli (see results of Experiment 3b). In this case, the discrimination of two linear dimensions would not be directly comparable with results of Experiment 1b. To unravel this problem, it becomes necessary to carry out an experiment that directly tests the cumulative area hypothesis, by verifying whether the snails discriminate discrete quantities, even when the cumulative area of two stimuli is equalled.

A second continuous quantity that snails could have used as a proxy of number is the convex hull of the group. In our experiment, the space between two adjacent bars was kept constant, and the convex hull increased with the number of elements in the group. Several species, including humans, have been observed to use convex hull as a proxy of number during quantity discrimination^56,57^. This issue can be experimentally addressed by testing an animal in a numerical task in which these non-numerical features are made irrelevant^58^.

Two other continuous cues, density and cumulative contour length (i.e. the sum of the perimeters of the items of the set), are potentially relevant for snail shelter choice. If two sets of objects occupy the same space (i.e. have the same convex hull), the more numerous also has a greater density. Density could thus be used in some conditions as a proxy of number^59,60^. In our experiment, density was kept constant, but this variable could be relevant whenever the subject is required to discriminate two different quantities with the same convex hull. In numerical experiments, the contour length strictly co-varies with surface area and the relative importance of these two variables has been rarely studied independently^61,62^. In the few cases in which this has been done, with the only exception of human infants, it has been found that subjects use the total area rather than the perimeter (Mosquitofish^57^; Chimpanzee^5^; Rhesus macaques^63^; Human infants^64^; Pigeons^65^). However, snails have a nervous system and a visual system markedly different from those of vertebrates and insects, and the situation may be different.

### Experiment 2: the influence of area, convex hull, and density on quantity discrimination

**Figure 4:**
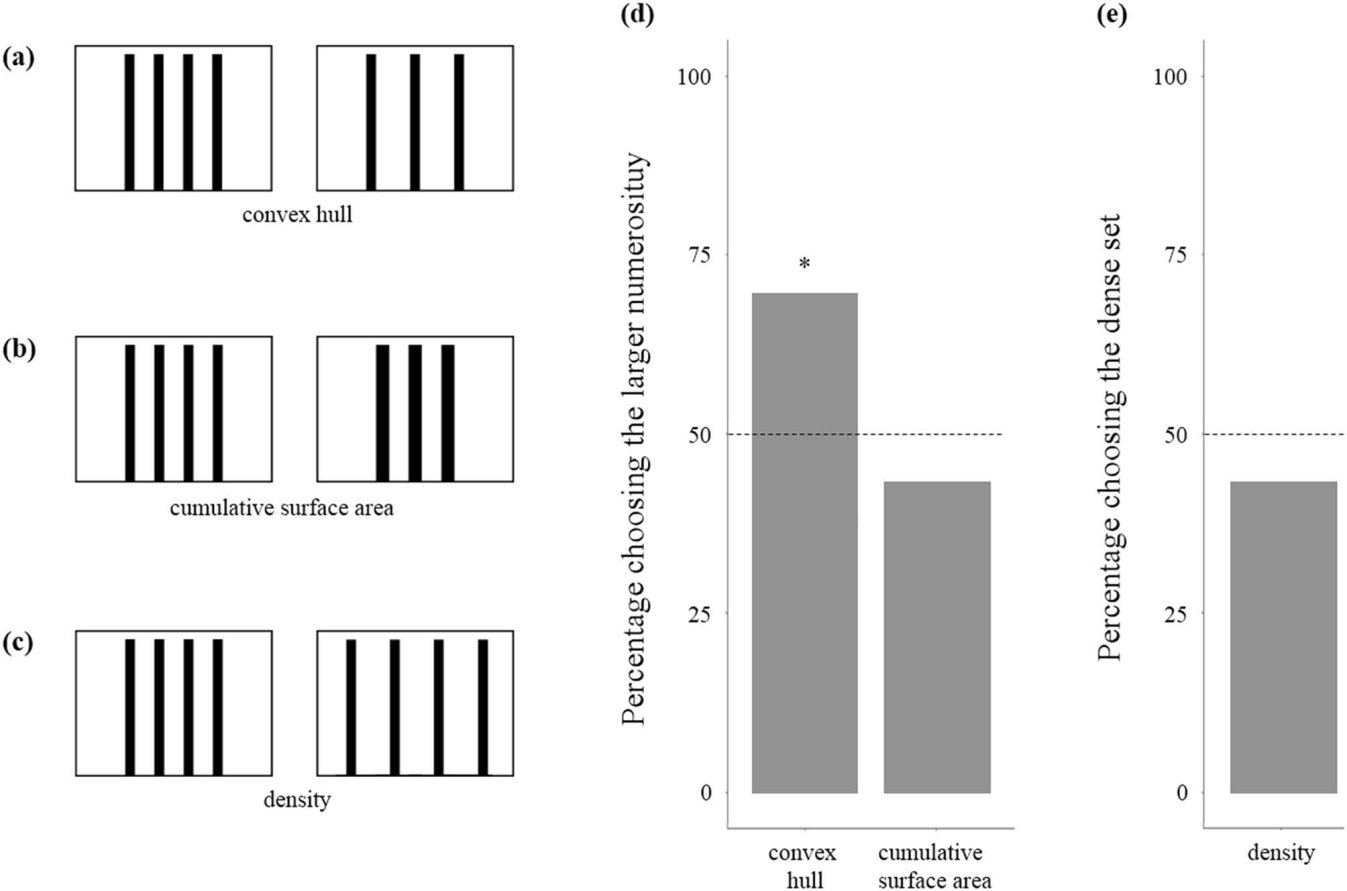
(**a**–**c**) Stimuli used in Experiment 2a, 2b, and 2c, respectively. (**d**) Percentage of snails choosing the stimulus with larger number of bars in Experiment 2a and 2b. When the stimuli were controlled for convex hull (Experiment 2a), snails showed a significant preference for the larger quantity (*P* = 0.028); when stimuli were controlled for the cumulative surface area, snails did not show any preference (Experiment 2b; *P* = 0.465). (**e**) When stimuli differed in density (Experiment 2c), snails did not show any preference (*P* = 0.465). Dotted line represents the expected preference by chance, and asterisks indicate significant deviations from the chance level (*P* < 0.05).

To study the influence of non-numerical variables on a snail’s preference, we presented the discrimination 3 versus 4, controlling the stimuli for convex hull (Experiment 2a; Fig. 4a) and for cumulative surface area (Experiment 2b; Fig. 4b). As side-effect of controlling for convex hull the two stimuli had a different density. To test if dune snails had any preference for dense or sparse clusters, we presented a discrimination between the same number of bars with a different density (Experiment 2c; Fig. 4c).

#### Results and discussion

In Experiment 2a, 21 out of 30 subjects chose the larger quantity controlled for convex hull, significantly above chance (*χ*^2^_1_ = 4.800, *P* = 0.028, *φ* = 0.283; Fig. 4d). In Experiment 2b, in which cumulative surface area was kept constant, the number of subjects choosing the larger quantity (13 out of 30) was not significantly different from chance (*χ*^2^_1_ = 0.533, *P* = 0.465, *φ* = 0.094). The Bayes Factor was 17 indicating that the hypothesis that snails did not prefer the larger quantity was much more likely than the alternative hypothesis. In Experiment 2c snails did not show a preference for dense or sparse bars (dense bars: 13 out of 30; *χ*^2^_1_ = 0.533, *P* = 0.465, *φ* = 0.094; Fig. 4e). The Bayes Factor was 17 indicating that the hypothesis that snails did not prefer the stimuli with larger density was much more likely than the alternative hypothesis.

The results of this experiment suggest that snails use continuous variables for their estimations of discrete quantities. When tested with stimuli in which the convex hull, i.e. a convex polygon enclosing all bars, was equalled, subjects continued to choose the larger quantity. Density of bars is however a cofounding variable in this experiment because as a by-product of controlling for convex hull, the two stimuli had different density. A preference for denser clusters instead of the discrimination of the larger numerosity could explain the preference for four bars we observed in this experiment. In Experiment 2c, we found no evidence that snails prefer denser to sparser clusters of bars, an indication that the convex hull is likely not a perceptual cue used by dune snail to choose their refuges.

Conversely, when the cumulative area of stimuli was paired, the choice of the larger numerosity disappeared, suggesting that dune snails use this variable to discriminate between quantities of bars. Cumulative surface area is probably the continuous variable most frequently used for numerical discrimination in the animal kingdom. Some species, apparently, do not process numerical information and use cumulative area as a proxy of number^54,66,67^. However, even those species that definitely have been shown to possess core numerical systems, including humans, preferentially use the cumulative area information to solve numerical tasks or combine numerical and continuous information to increase accuracy^8,25^. The teleost *Gambusia holbrooki*, for example, can solve numerical tasks using only number or only area, but the performance improves when it is allowed to use both^25,57^.

Therefore, it is quite plausible that dune snails too, when orienting towards the largest group of bars, are selecting the largest cumulative area, or even more simply, they are guided by a scototactic response towards the darkest part of the landscape^41^. Before accepting the hypothesis that this species uses the cumulative area to choose the larger set of bars, it is necessary to exclude two other hypotheses that are compatible with the above results. The first concerns the fact that, in the above experiments, we used stimuli with widths just above the perception threshold (see Experiment 5b). Even if we showed they can perceive an isolated bar 2-cm wide, it might be difficult for snails to precisely distinguish just-above-threshold bars when grouped together, and hence, they may be prompted to rely on the amount of visible surface. This issue was addressed in Experiment 3.

A second important factor concerns the fact that, to control for surface area in Experiment 3b, we used bars of different widths. If snails have spontaneous preferences for the width of the bars, this could affect the results. For example, larger stems may represent refuges that are more valuable because they hide snails from predators better. Another reason for preferring wider bars is that terrestrial gastropods likely lack efficient mechanisms of depth perception: wider stems are probably perceived as closer and, hence, faster to reach^68,69^. Consequently, in Experiment 3b, snails could have been attracted to the cluster containing wider bars, counterbalancing their preference for larger clusters. This issue was addressed in Experiment 4.

It is noteworthy that even if we could dismiss the role of cumulative surface area, there is a fourth important continuous variable, which is cumulative contour length, that in Experiment 1 increases linearly with numerosity ratio and that snails could have used as a cue in quantity discrimination. We will consider this problem in Experiment 3 and 4.

### Experiment 3: influence of bar width

**Figure 5:**
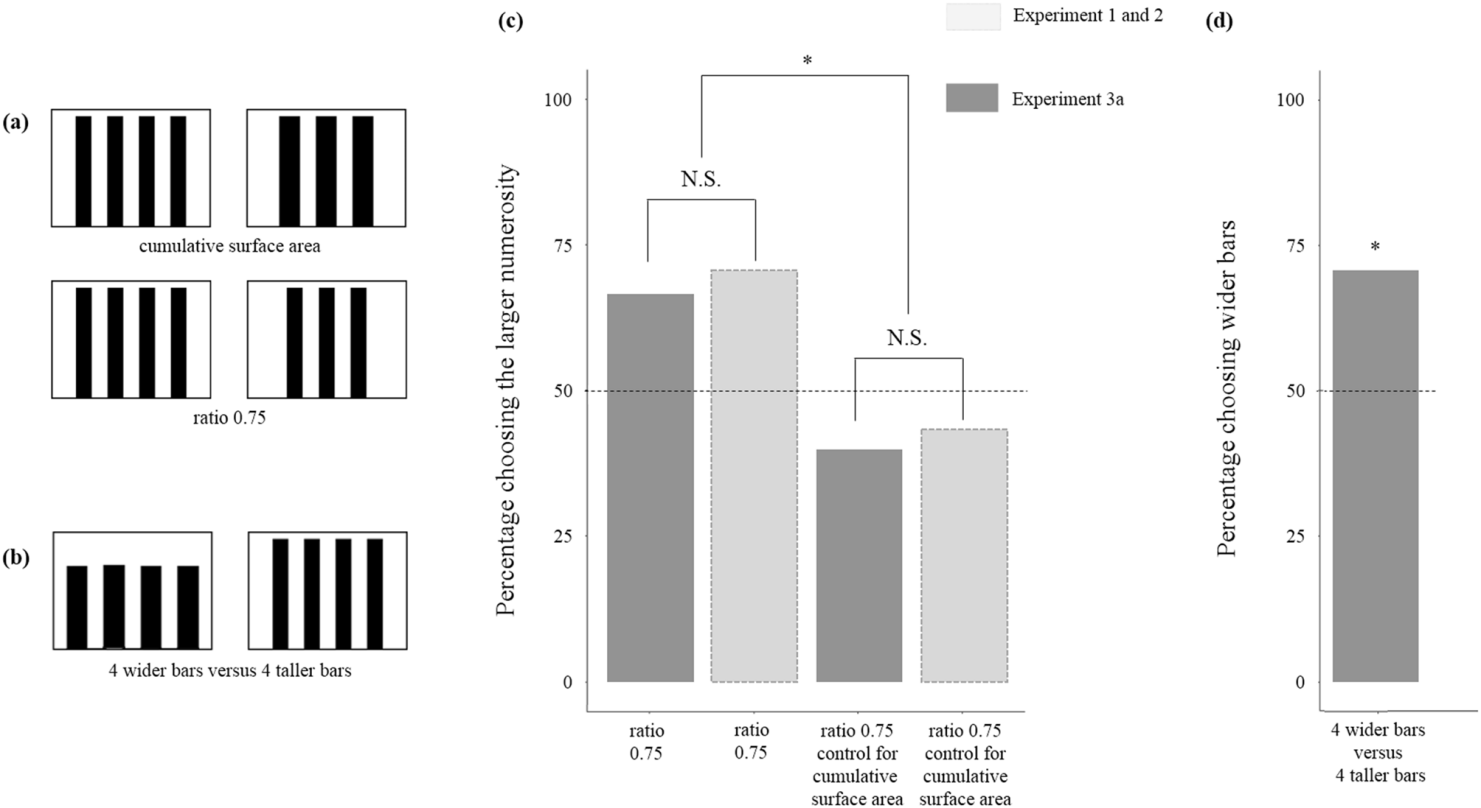
(**a**,**b**) Stimuli used in Experiment 3a and 3b, respectively. (**c**) Percentage of snails choosing the stimulus with larger number of bars in Experiment 1 and 2b (light grey) and Experiment 3a (dark grey). An overall analysis showed a significant effect of experimental condition (*P* = 0.004), but no effect of the width of stimuli (*P* = 0.679), nor interaction (*P* = 0.943). (**d**) In Experiment 3b, snails showed a preference for the stimulus with wider bars (*P* = 0.029). Dotted line represents the expected preference by chance, and asterisks indicate significant deviations from the chance level (*P* < 0.05).

To exclude that the use of bars just above-threshold could have determined poor discriminability, in Experiment 3a, we repeated the discrimination 3 versus 4 shown in Experiment 1b and Experiment 2b, but we used stimuli doubled in width (Fig. 5a). To verify if snails have an innate preference for wider stems in Experiment 3b, we gave snails the choice between bars of different widths, while keeping the number of elements and the cumulative surface area in the two clusters identical (Fig. 5b).

#### Results and discussion

When stimuli were controlled for overall surface area (Experiment 3a), snails continued to manifest no preference for the larger numerosity, even when bars were doubled in width (12 out of 30 subjects, *χ*^2^_1_ = 1.200, *P* = 0.273, *φ* = 0.141; Fig. 5c). The group of snails tested with stimuli non-controlled for area showed a tendency to prefer the larger quantity though the preference did not reach significance (20 out of 30 subjects, *χ*^2^_1_ = 3.333, *P* = 0.068, *φ* = 0.236). The difference between the two groups is significant (*χ*^2^_1_ = 4.339; *P* = 0.037, *φ* = 0.269). We performed an overall analysis comparing the results with 4 cm-wide bars of this Experiment with the same types of experiments done with 2 cm-wide bars (Experiment 1b: 3 vs. 4 non-controlled, Fig. 3a; Experiment 2b: 3 vs. 4 controlled for area, Fig. 4b) using a GLM model. We found a significant effect of experimental condition, controlled versus non-controlled (*χ*^2^_1_ = 8.497, *P* = 0.004), no effect of the width of stimuli (*χ*^2^_1_ = 0.171, *P* = 0.679), and no interaction (*χ*^2^_1_ = 0.005, *P* = 0.943, *R*^2^ = 0.097). The approximate Bayes factor was 104 indicating that the GLM model without the effect of the factor “width” was much more likely to explain the performance of the subjects than the model with such effect. In Experiment 3b, 21 out of 30 subjects chose the stimulus with wider bars (*χ*^2^_1_ = 4.800, *P* = 0.029, *φ* = 0.283; Fig. 5d).

Replication of the Experiments 1b and 2b using bars doubled in width confirmed the original results, therefore excluding that the outcome the experiment controlling for cumulative surface area (Experiment 2b) was the artefactual consequence of using unsuitable stimuli.

In Experiment 3b, we found a clear preference for wider bars, even if the number of bars and their cumulative areas were the same on the two sides. In the environment in which dune snails live, stems show little variation in diameter. To an animal that lacks independent mechanisms for estimating the distance of an object, a bar twice in width likely appears much closer than the thinner one. The results of this experiment allow an interpretation of the results of Experiment 2b, which does not imply that snails are basing their quantity discrimination on the cumulative surface areas of the stems.

To summarise, there are two main hypotheses to explain the results so far obtained. The first hypothesis suggests that snails are able to discriminate between different amounts of stems based only on the areas occupied stimuli. This hypothesis is plausible because Zanforlin has shown that *T. pisana* spontaneously orient towards the larger of two dark surfaces and we confirmed this finding^41^. At this stage, a second hypothesis is equally plausible, i.e. that snails possess more sophisticated perceptual and cognitive functions, capable of extracting more detailed information about the characteristics of the surrounding environment. These putative functions could even include the possibility of snails to possess a true numerical system, i.e. a system capable of extracting information about the number of objects in a set, regardless of the other cues that co-vary with number.

However, there is no single experiment capable of directly contrasting the two hypotheses. We can only perform experiments to test some of the predictions generated by these hypotheses. First, the hypothesis of snails being guided only by the cumulative area of bars predicts that, in a choice test, the attraction to one stimulus is determined only by the amount of black surfaces and that the degree of attraction should be independent from the shape or orientation of the elements in the set. To verify it, we tested the snails in a choice between the same set of bars but with different orientations (Experiment 4a). Second, if the result of Experiment 2b was due to a preference for larger bars and not to a preference for larger cumulative surface area, snails should still prefer the larger numerosity when the control for cumulative areas is obtained by manipulating the heights instead of the widths of the bars in the sets. We verified this hypothesis in Experiment 4b.

As previously mentioned, cumulative contour length is another continuous variable that varied linearly with numerosity ratio in our stimuli. By, studying the preference between two shapes, Zanforlin found that dune snails were attracted to figures with the longer perimeter^41^. He considered this effect to be of secondary importance since he observed that snails oriented themselves towards the figure with the longer perimeter if the two figures had the same area, but preferred a figure with shorter perimeter if this had a larger area. In Experiment 3b the two stimuli had the same area whereas the ratio of the perimeters was 0.82 and the snails significantly preferred the stimulus with the smaller perimeter. This suggests that they do not rely on the perimeter at least when they must, as in this study, compare two groups of figures.

### Experiment 4: further tests of the cumulative area hypotheses

**Figure 6:**
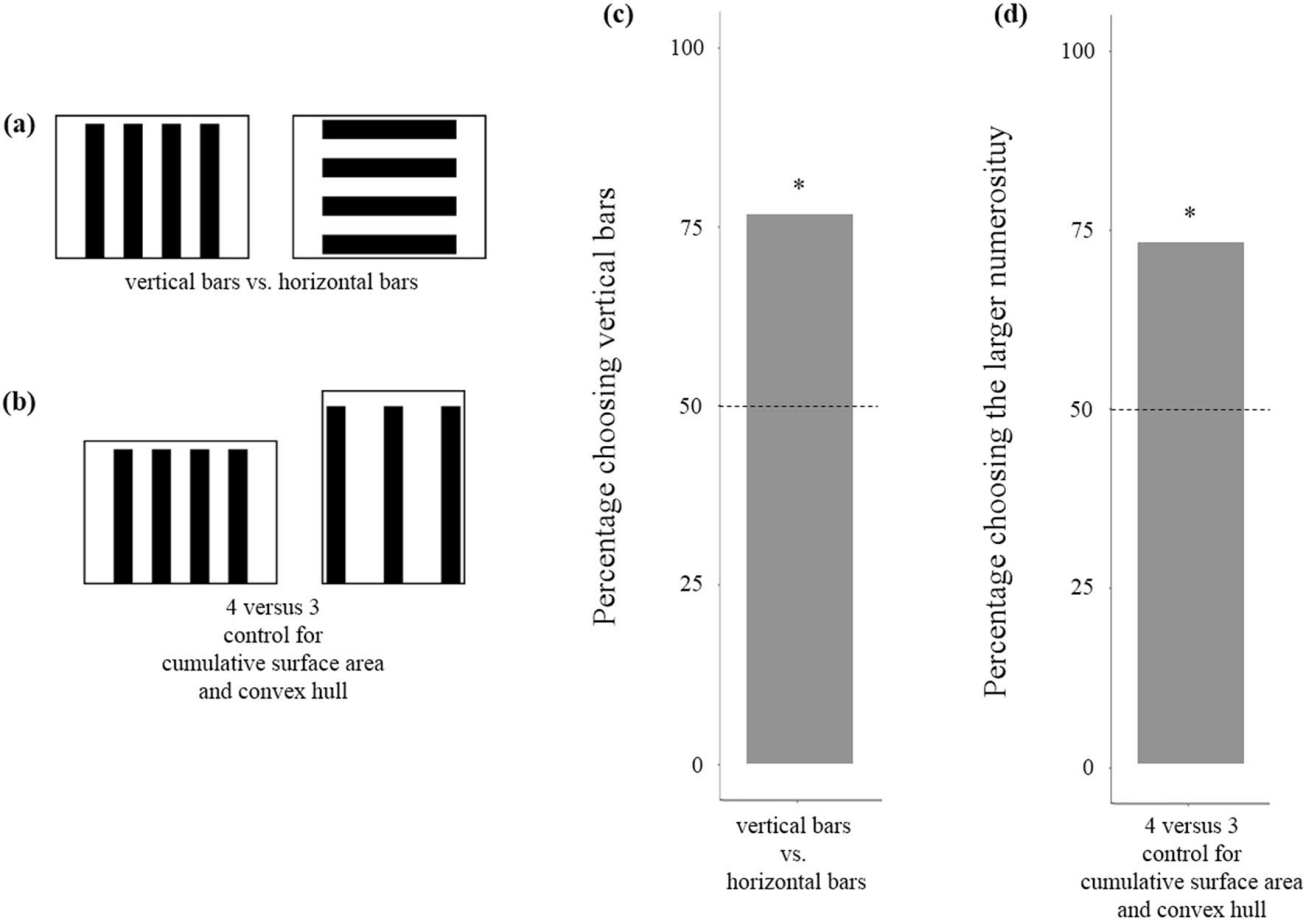
(**a**,**b**) Stimuli used in Experiment 4a and 4b respectively. (**c**) In Experiment 4a, snails showed a significant preference for the stimulus with vertical bars (*P* = 0.004). (**d**) In Experiment 4b, snails showed a significant preference for the stimulus with larger numerosity (*P* = 0.011). Dotted line represents the expected preference by chance, and asterisks indicate significant deviations from the chance level (*P* < 0.05).

In Experiment 4, we checked whether the behaviour of dune snails could simply be explained by a scototactic reaction, i.e. an attraction to the larger amount of “black”. In Experiment 4a, subjects were given the choice between the same number of bars, but with different orientations (horizontal vs. vertical; Fig. 6a). As height, width, and cumulative surface area were identical, an animal using a scototactic response should not discriminate among them. In Experiment 4b, we controlled stimuli for cumulative areas and convex hull but the area control was obtained by manipulating the heights instead of the widths of the bars (Fig. 6b). If dune snails use area as a proxy of number, they should not discriminate between the two stimuli. If the outcome of Experiment 2b was due to spontaneous preference for larger stems, in this experiment they should prefer the larger quantity of bars.

#### Results and discussion

In Experiment 4a, the number of subjects that chose the stimulus with vertical bars (23 out of 30) was significantly above chance (*χ*^2^_1_ = 8.533, *P* = 0.004, *φ* = 0.377; Fig. 6c). In Experiment 4b, the number of snails that chose the larger quantity (22 out of 30) was significantly above chance (*χ*^2^_1_ = 6.533, *P* = 0.011, *φ* = 0.330; Fig. 6d).

The two stimuli used in Experiment 4a had the same cumulative surface area, heights, and widths. Preference for the stimulus with vertically oriented bars, therefore, is not compatible with a simple response to the amount of “black” (i.e. a scototactic response) and requires that such a mechanism is paired with a detector of orientation. In Experiment 4b, we found that dune snails continued to prefer the larger quantity, even with equaled cumulative surface area, provided this was obtained by manipulating the heights instead of the widths of the bars. With the results of Experiment 3b, this finding supports the hypothesis that, in Experiment 2b, subjects failed to choose the larger quantity, not because the cumulative area was equaled in the two stimuli, but because wider bars attracted them.

### Experiment 5: reliability and repeatability

**Figure 7:**
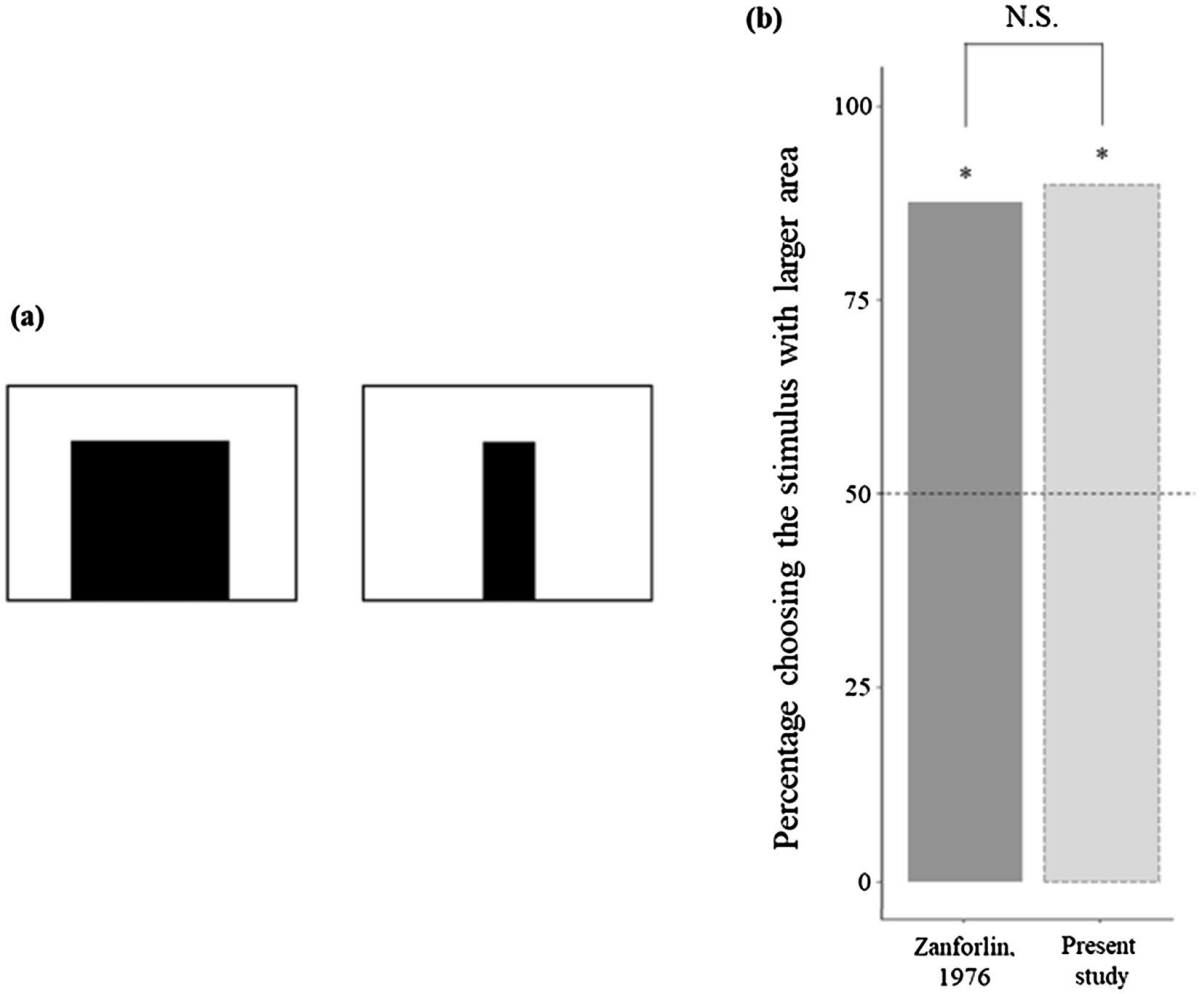
(**a**) Replication of one experiment conducted by Zanforlin^41^. (**b**) Percentage of snails choosing the larger stimulus shown in the previous study^41^ (dark grey) and in the current study (light grey). Preference did not differ between the two studies (*P* = 0.803). Dotted line represents the expected preference by chance, and asterisks indicate significant deviations from the chance level (*P* < 0.05).

**Figure 8:**
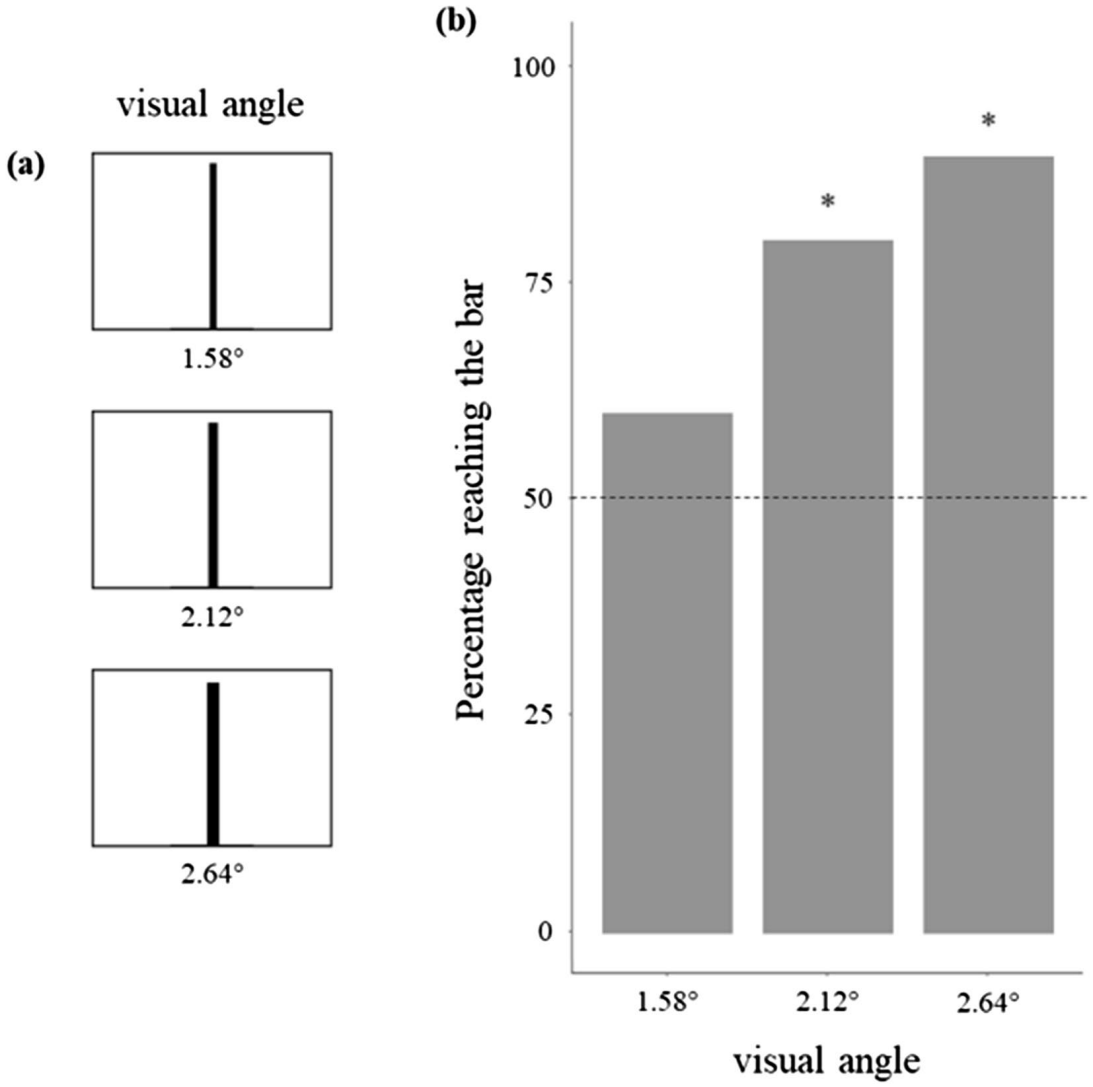
(**a**) Stimuli used in Experiment 5b. (**b**) Percentage of snails reaching the bar in Experiment 6b. Snails showed a preference for the bar with 2.12° visual angle (*P* = 0.007) and 2.64° (*P* < 0.001), not for bar with 1.58° visual angle (*P* = 0.371). Dotted line represents the expected preference by chance, and asterisks indicate significant deviations from the chance level (*P* < 0.05).

Four additional tests were performed to measure repeatability and reliability and to set the width of stimuli used. In Experiment 5a, we replicated one of the original experiments of the study of Zanforlin (Fig. 7a) to assess the robustness of the procedure when using a different population and after some changes in the setup, particularly to the type of light and the material that we used for building the arena^41^. There are no data on visual acuity of *T. pisana*. Zanforlin tested snails with bars 2 cm versus 3 cm in width (approx. 2.2° and 3.3° respectively), finding a preference for the latter stimulus^41^. Experiment 5b was aimed at verifying the minimum width necessary for a stimulus to elicit an approach response in the conditions of our experiment (Fig. 8a). Experiments 6c and 6d were aimed respectively at the reliability and repeatability of our procedure.

#### Results and discussion

In Experiment 6a (inter-study repeatability), 20 out of 24 subjects chose the stimulus with larger surface area (*χ*^2^_1_ = 10.667,* P* < 0.001, *φ* = 0.471). In previous work, 30 out of 35 subjects chose the stimulus with larger surface area^41^. Using a GLM model, we found no difference between the two studies (*χ*^2^_1_ = 0.062,* P* = 0.803, *R*^2^ = 0.002; Fig. 7b).

In Experiment 5b (minimum discriminable width), 12 out of 20 snails chose the bar that corresponded to a 1.58° visual angle (*χ*^2^_1_ = 0.800, *P* = 0.371, *φ* = 0.141), 16 out of 20 the bar with the 2.12° visual angle (*χ*^2^_1_ = 7.200, *P* = 0.007, *φ* = 0.424), and 18 out of 20 the bar with the 2.64° visual angle (*χ*^2^_1_ = 12.800, *P* < 0.001, *φ* = 0.567, Fig. 8b).

In Experiment 5c (inter-rater reliability), the binary measure of the snail’s choice did not differ between the two scorers (98.2% concordance, Cohen’s kappa = 0.96). In Experiment 5d (intra-study repeatability), the preference for the larger quantity in the replicate experiment was 19/24 (*χ*^2^_1_ = 8.167, *P* = 0.004, *φ* = 0.413), whereas in the original experiment it was 17/24; the difference was not significant (*χ*^2^_1_ = 0.446,* P* = 0.504, *φ* = 0.096).

Zanforlin described a tendency to reach for dark shapes and prefer larger stimuli in a *T. pisana* population from the north Italy coast^41^. Our subjects were collected approx. 65 km northeast along the coast in a more anthropised area. Inter-population differences in behaviour have been reported for many species, including snails^70^. With Experiment 6a, we showed that the same behaviour is present in snails from the population we studied. The response thus appears robust to changes in some details of the apparatus and procedure, including the use of a different source of illumination. Experiment 6c and 6d show that the response of dune snails in our discrimination tests is also robust and that its measure is highly reliable and replicable.

Prior to the start of the main experiments, in Experiment 6b, we determined the minimum visual angle to elicit an approach response from snails. With a bar width of 1.10 cm (1.58° visual angle), snails showed a random response, whereas they were attracted by the stimulus with bars of 1.48 and 1.84 cm (2.12° and 2.64°, respectively). For Experiments 1 and 2, we therefore adopted 2 cm wide bars corresponding to an angle of 2.86°.

## General discussion

The results of this study indicate that *Theba pisana* orients preferentially towards clusters of stems and can excellently discriminate the cluster with the larger quantity of stems. The maximum discrimination of quantities that we have observed is 4 versus 5 items, an acuity only exceeded by a few mammals and birds and one species of fish^53^. There is a general trend in the literature of more advanced cognitive capabilities being observed in species provided with a large and complex nervous system^49,71,72^. However, in some cases, ecological adaptations can drive the evolution of extraordinary cognitive capacities, as in the examples of spatial memory in food-storing birds or homing in pigeons^73,74^. Indeed, good numerical capacities have been found in a few cases in small-brained animals too (e.g., guppies^11^ or honeybees^32^). As mentioned before, during sunny days, the failure to find an adequate shelter will almost inevitably lead a dune snail to death, a selective force that certainly may have favoured the evolution in this species of an elaborate cognitive mechanism for selecting the most favourable shelter condition.

Which type of information are dune snails using in choosing groups of shelter? Many species, including humans, routinely estimate numerosity using non-numerical information that co-varies with number, such as the amount of movement, the cumulative surface area, or the convex hull of a set of objects^75^. This may be expected to occur even more frequently in organism such as gastropods that are provided with a relatively simple visual system^68,76^. For example, in our study snails could have discriminated quantities relying on the convex hull given that in Experiment 1 this quantity increased linearly with the amount of bars. Experiment 2a showed that when the convex hull was paired between stimuli, snails still preferred the stimulus with the larger numerosity. However, density was a potential confounding variable of this experiment. An influence of the density of bars cannot itself explain the results of Experiment 1 but in Experiment 2a the set with four bars had a greater density than the set with three and hence the choice of the former could be explained by a preference for denser stimuli. This possibility was excluded in Experiment 2c during which snails shoved no tendency to prefer clusters with denser bars.

Another good candidate is cumulative contour length, since it also increased linearly with the number of bars. Human infants and possibly honeybees rely on differences in contour length to estimate quantities^30,64^. When studying the preference between two shapes, Zanforlin found that snails preferentially oriented themselves towards figures with a longer perimeter^41^. He found this preference to be weak since snails preferred figures with a larger area to figures with a longer perimeter. In addition, the capacity to discriminate a figure with a longer perimeter does not automatically imply that snails are also able to calculate the cumulative contour length of a set of figures and compare it with the cumulative contour length of another set. In order to explain Experiment 1 with the use of contour length, the snails must be quite accurate in this capacity, i.e. snails are able to discriminate two cumulative contours that differ by a 0.80 ratio. Given the nature of the stimuli used in this study, it would be extremely problematic to do an experiment in which this variable is controlled without varying also the area or width of the stimuli (see below) or using stimuli with very different shapes. Indeed, some of the experiments of this study make it unlikely that snails could have used the cumulative contour as a cue to discriminate quantities. In Experiments 2b and 3a for example, cumulative contour differed by 0.77 ratio but the set with the longer contour was not preferred. In Experiment 4b snails preferred the set with the larger numerosity even if the two set had almost the same contour length (0.97 ratio) and in Experiment 3b the contour ratio is 0.82 and snails prefer the stimulus with the smaller contour.

In the species studied so far, cumulative surface area is the non-numerical cue most frequently used to discriminate different quantities of objects. The possibility that dune snails were using this cue is supported by Experiment 1c in which we found that the discrimination of two areas closely mirrors the discrimination of number of bars. In this experiment, we found that for a given ratio, snails were slightly less accurate in discriminating areas than they were in discriminating the number of bars in Experiment 1a and 1b. However, this small difference in acuity could be due to the fact that in the area discrimination experiment we did not use stimuli that resemble the stem that snails climb in nature. Indeed, when the area hypothesis was directly tested (Experiment 2b) we observed that the ability to discriminate two quantities disappeared. Usually, this kind of results would indicate that an animal is primarily or exclusively using non-numerical cues to estimate quantity^7,23^. In the case of *T. pisana*, however, there is an alternative explanation for this result. Because of control of the cumulative area, stimuli with smaller numbers contained wider bars. If dune snails have a spontaneous preference for wider bars, this could have offset their preference for the larger clusters. In nature, there are various reasons to prefer large stems; for example, due to their size, they can better support the weight of snails or better hide them from predators. Another explanation is that since terrestrial gastropods likely lack an efficient depth estimation system^68,69^, larger stems will appear closer and hence faster to reach. Indeed, a strong preference for wider bars was confirmed in Experiment 3b, which leaves room for different interpretations of Experiment 2’s results. This issue is not easy to solve and is representative of the difficulties often encountered in assessing the role of non-numerical variables in quantity discrimination^7,8,25^. For example, some studies have exploited the preference for the number of food items or of the number of group mates. In such experiments, it is difficult to manipulate variables such as cumulative surface area because there are often preferences for food items or for conspecifics of a certain size^20,61^. There are difficulties also in using artificial stimuli, such as in experiments that employ operant conditioning. A change in one non-numerical variable often affects other variables and some, such as area and contour or density and convex hull, strictly co-vary so that it is generally problematic to test their effects independently^50,57^.

Similarly, there is no single decisive experiment that can prove or disprove that snails have used the cumulative surface area to solve Experiment 1. Zanforlin suggested that orientation preferences exhibited by dune snail could simply be explained by a scototactic response, i.e. an attraction to the largest dark area, irrespective of its shape^41^. Two experiments ruled out this hypothesis in the case of selection of the larger quantity of stems. In Experiments 4a (vertical vs horizontal bars) and 4b (area equated increasing the height) the two stimuli had an identical cumulative surface area, but the subjects chose respectively the stimulus with the correct orientation and with the larger number of elements. However, we cannot exclude that snails use the area information in a more complex way. For example, a preference for the larger cumulative surface coupled with detector of “vertical bars” and with a preference for larger bars could explain all of our results.

Other hypotheses are equally conceivable at this stage of the research. For example, snails may be able to directly estimate the number of bars present in their visual field. A functions such as numerical discrimination, which is apparently complex, may actually be mediated by relatively simple neural circuits, as artificial neural network studies seem to indicate^77^. Indeed in fish, discrimination of numerosity does not seem to be more cognitively demanding than discrimination of continuous dimensions such as area or length and it was found to appear earlier in development^26,78,79^. In support of this view, some studies suggest that in humans, numerosity information is extracted at the early stages of analysis of visual input and can thus be considered a primary perceptual attribute like shape or colour^80^.

In sum, our experiments did not reach a firm conclusion about which cue was used by snails in the discrimination of number of refuges. Dune snails can discriminate between two objects that differ in area (Experiments 5 and 6b; Zanforlin^41^) or in contour length^41^, however this study indicates that neither of these two capacities seems sufficiently accurate to allow the discrimination of quantities shown in Experiment 1. Our experiments also seem to rule out the possibility that they base their choice on density or convex hull. However, we do not have even a direct proof that they used the numerical information to solve the tasks. In fact, other hypotheses could be advanced, for example that snails use perceptual cues not considered here, or that they use a combination of different cues as suggested for humans^81,82^, or that they integrated numerical and non-numerical information as most vertebrates do^26,83,84^. At least with the spontaneous preference paradigm adopted here, it is hard to devise experiments that could circumvent the methodological problems previously highlighted and disentangle these alternatives. However, gastropods are capable of associative learning, and it might be possible to study numerical discrimination using discrimination learning paradigms similar to those employed in the study of vertebrate species^85,86^. Numerical abilities are not a prerogative of animals with large and complex nervous systems and there is convincing evidence that such capacity is present in at least one invertebrate species, the honeybee^28,30^. A challenge for future investigation will be to determine whether the capacity to process numerical information is limited to social insects or it is present in other invertebrate taxa.

As a final remark, one might argue that, from an evolutionary point of view, it is irrelevant whether an animal enumerates objects or instead simply uses a proxy of number to solve an ecological problem and that consequently whether an animal is endowed with a numerical system only matters to cognitive science. In some cases, though, the quality of information acquired may matter to evolution. Behavioural ecology is rich with examples in which animals need to acquire precise countable information to make optimal decisions. Kin selection theory, for example, predicts that to make an appropriate decision, an actor needs to precisely evaluate the number of relatives that would benefit from an altruistic act^87^. Pied flycatchers returning from wintering areas inspect the nests of great tits and use information about the number of eggs laid to estimate the quality of the breeding site^88^. When several sticklebacks compete in patchy environments, they distribute themselves so as to minimise competition and maximise their feeding rate^89^. In general, optimality models assume that individuals acquire information with infinite precision, but this assumption has rarely been verified^90^. Conversely, there is evidence that the way in which information is acquired, processed, and stored can influence the outcome of evolutionary processes^91,92^. In particular, the adoption of rules of thumb can decrease decision time and reduce the need for neural computation, but in many cases it may lead to inaccurate assessment and ultimately to suboptimal decisions^93^.

## Material and methods

### Ethical statement

The current legislation of our country (Italy) permits experiments that consist of observation of invertebrates’ behaviour without any approval by an ethical committee. However, all subjects were maintained and tested following the ASAB/ABS Guidelines for the Use of Animals in Research^94^.

### Subjects

We collected adult *Theba pisana* (shell diameter varying from 13 to 18 mm) from the sand dunes near the mouth of Piave river (Jesolo, northern Italy 45°31′46.5″N, 12°43′32.7″E). Subjects were collected from July to September according to Italian law (BUR n. 47/1974). In the laboratory, they were housed in plastic boxes (40 × 65 × 30 cm) that were covered with a net to permit air circulation and moistened at regular intervals. Each maintenance box was provided with sand and dry grass. Snails were fed three days a week with commercial lettuce. The temperature was kept at 25 °C ± 1, and light was provided from a 30-W fluorescent lamp (12:12 light/dark period).

In each experiment, we considered only subjects that actively reached one of the two presented stimuli. When subjects did not reach one stimulus within the predetermined time (see details below), we discarded and substituted them with new subjects of the same group. We tested 164 snails in Experiment 1 (24 for each discrimination), 90 snails in Experiment 2 (30 for each discrimination), 90 snails in Experiment 3 (30 for each discrimination), 60 snails in Experiment 4 (30 for each discrimination), and 108 snails in Experiment 5 (24 for Experiment 5a and 5d, 20 for each discrimination in Experiment 5b) for a total of 702 snails. To avoid the influence of experience, each subject was tested once; after the experiment, snails were kept in a separate maintenance box until they were released at the site of capture.

### Apparatus

The experimental apparatus was similar to that used by Zanforlin^41^. It consisted of a white plastic circular arena (diameter 80 cm, height 75 cm; Fig. 1b) placed in a complete dark room. The arena was uniformly lit by a *LED* lamp (450 lumens, opening angle 100) placed 100 cm from the floor.

The stimuli were single black shapes or groups made with Microsoft Word. All stimuli were printed on A3 white paper with a laser printer (Kyocera TASKalfa 4052ci). Stimuli varied in number, size or density, depending on the experiment (see details below). During the test, the two stimuli were placed on the walls of the arena. The centre of the two printed papers formed an angle of 60° with the centre of the arena; this setup allowed snails to see both stimuli at the same time. Previous studies reported that snails can detect different stimuli in a 180° visual field^41^. The relative left–right position and the position of stimuli in the arena were randomised for each subject.

### Procedure

We performed the experiments between 07:00 and 13:00, the period of maximal activity of snails in the laboratory. The experimenter collected one active subject and positioned it in the centre of the arena. The trial ended when the subject reached the wall of the arena where the stimuli were located. A choice was considered when the subject touched one of the two papers where the stimuli were printed. We allowed 20 min for the subjects to reach one stimulus. Snails that did not move or did not reach one stimulus within this interval were discarded and replaced with another individual to maintain the predetermined sample size. After each trial, we cleaned the arena with 99% ethanol to remove any chemical cues.

### Experiment 1: discrimination of the quantity of refuges

In Experiment 1a, each snail was given the choice between a single black bar and a group of three bars. All bars were of the same surface area (width 2 cm, height 28 cm) and separated by 4 cm in the groups (Fig. 2a). In Experiment 1b, dune snails were given the choice between two groups of identical bars that differed in numerosity. We carried out five comparisons: 4 versus 1, 4 versus 2, 4 versus 3, 4 versus 5, and 5 versus 6 (ratios of 0.25, 0.50, 0.75, 0.80, and 0.83, respectively). As in Experiment 1a, we used 2 × 28 cm black bars separated by 4 cm (Fig. 2b). In Experiment 1c, we presented five discriminations with the same area ratios used in Experiment 1: 0.25, 0.50, 0.75, 0.80, and 0.83. In each comparison, one stimulus was a 14 × 14 cm black square; the second stimulus varied in surface area in relation to the ratio (0.25: 7 × 7 cm; 0.50: 9.89 × 9.89 cm; 0.75: 12.12 × 12.12 cm; 0.80: 12.52 × 12.52 cm; 0.83: 12.75 × 12.75 cm; Fig. 3c). We tested 24 subjects for each discrimination.

### Experiment 2: the influence of area, convex hull, and density on quantity discrimination

In Experiment 2a, to control for convex hull, we presented a discrimination between one set of four 2 × 28 cm bars separated by 4 cm and a second set of three 2 × 28 cm bars separated by 7 cm so that width and height were identical in the two stimuli (20 × 28 cm; Fig. 4a). In Experiment 2b (control for cumulative surface area), we presented a discrimination between a set of four 2 × 28 cm bars separated by 4 cm and a second set of three 2.67 × 28 cm bars separated by 4 cm so that the cumulative black area (224 cm^2^) was identical in the two sets of stimuli (Fig. 4b). In Experiment 2c, to control for density, we presented a discrimination between one set of four 2 × 28 cm bars separated by 4 cm and a second set of four 2 × 28 cm bars separated by 7 cm (the same inter-item distance used in Experiment 2a). We tested 30 subjects for each discrimination.

### Experiment 3: influence of bar width

In Experiment 3a, we investigated the effect of bar width on snails’ preferences regarding numerosity when sets of stimuli were controlled for the cumulative area. We tested 30 subjects for their preference between a set of four 4 × 28 cm bars separated by 4 cm and a set of three 5.33 × 28 cm bars separated by 4 cm (Fig. 5a). As a control, we tested 30 additional subjects in a 3 vs 4 discrimination non-controlled for cumulative area (a replica of the 3 vs 4 discrimination of Experiment 1b, but using bars doubled in width); one stimulus was made of four 4 × 28 cm bars separated by 4 cm, and the other of three 4 × 28 cm bars separated by 4 cm (Fig. 5a).

In Experiment 3b, we investigated the snails’ spontaneous preferences on bar width. We presented to 30 subjects a discrimination between four 4 × 28 cm bars separated by 4 cm and four 5.3 × 21 cm bars separated by 4 cm (0.75 ratio). Bars in the two stimuli had the same area, 448 cm^2^, but were wider and shorter in one stimulus (Fig. 5b).

### Experiment 4: further tests of the cumulative area hypotheses

In Experiment 4a, 30 subjects were given a choice between two stimuli of the same size (28 × 28 cm), the same number and size of elements (four 4 × 28 cm bars separated by 4 cm), and the same cumulative surface area. In one stimulus the bars were vertical. The second stimulus was simply obtained by a 90° rotation of the first stimulus (Fig. 6a). In Experiment 4b, 30 subjects were given the choice between four 4 × 28 cm bars separated by 4 cm and three 4 × 37.3 cm bars separated by 4 cm (Fig. 6b).

### Experiment 5: reliability and repeatability

The apparatus and procedure used for these four experiments were the same as those described for Experiment 1. Experiments 5a and 5b were performed before the main experiments. In Experiment 5a (inter-study repeatability), 24 subjects were allowed to choose between two stimuli that differed in shape and area: a 30 × 30 cm black square and a 30 × 10 cm (height x width) black rectangle (Fig. 7a). Both stimuli were printed on A2 paper. In Experiment 5b (minimum discriminable width), we tested stimuli of three different widths. The first was a 1.10 × 28 cm black bar (corresponding to a 1.58° visual angle when the snail was in the centre of arena), the second a 1.48 × 28 cm black bar (2.12°), and the third a 1.84 × 28 cm black bar (2.64°). Each stimulus was printed on A4 paper with a vertical orientation and paired to a completely white A4 paper sheet with the same orientation (Fig. 8a). The criterion for choice was if the snail reached the base of one of the two sheets. For each type of stimulus, we tested 20 subjects. In Experiment 5c (inter-rater reliability), a second observer blind to the aims of the experiments reanalysed the videos of 56 randomly chosen subjects. In Experiment 5d (intra-study repeatability), a second experimenter blind to the aims of the experiments tested 24 individuals in one of the comparisons of Experiment 1 (3 vs 4), using the same procedure and apparatus.

### Statistical analysis

Statistical analysis was performed in RStudio version 1.2.5019 (RStudio Team (2015). RStudio: Integrated Development for R. RStudio, Inc., Boston, MA, URL http://www.rstudio.com). Statistical tests were two-tailed, and significance thresholds were *P* = 0.05. The effect size values (i.e., Phi coefficient *φ*, and *R*^2^) were reported for appropriate statistical tests.

We estimated the sample size necessary to achieve 90% power to be 21, setting significance level to .05, and proportion of preference to .85 (estimated from the first two experiments of this study, 5a and 1a. Accordingly, in Experiments 1 we used a sample size of n = 24 for each ratio tested and we used a sample size of n = 30 for all the other experiments.

For each discrimination, we compared the observed proportion of snails that chose the larger stimulus (calculated as the number of snails that chose the larger stimuli divided by the total number of snails that chose a stimulus) with that expected by chance (probability of random choices equals 50%).

For experiments with different quantity ratios (Experiments 1) or experiments in which we compared different conditions (Experiments 3a and 5a), we analysed the binary choice of subjects with a GLM (“glm” function from the “lme4” R package) with binomial errors distribution and the logit link function. We fitted the model with ratio (Experiments 1b and 1c) or width of stimuli (“width”, Experiment 3a) or type of experiment (Experiment 5a) as fixed factor to test differences due to the condition considered according to the experiment. We evaluated the effect of the parameters using the ‘Anova’ function of the ‘CAR’ R package.

In Experiment 3a, we calculated the approximate Bayes factor from the Bayesian Information Criteria to compare models with and without the type of stimulus (i.e., bar width) as a factor^95^. This method provides an approach to interpret non-significant results which is robust to small sample size^96^. For example, Bayes Factor’s values ≥ 10 indicate a strong similarity in the preferences between Experiments 1b and 1c, even though the type of stimulus differed between the experiments^97^. The Bayes Factor was calculated also for Experiments 2b and 2c in which we found non-significant results.

## Supplementary Information

Supplementary Video 1.

Supplementary Video 2.
